# Adherence to post-therapeutic multidisciplinary tumor board recommendation and its influence on oncological outcomes in high-risk prostate cancer patients following radical prostatectomy

**DOI:** 10.1007/s11255-025-04620-0

**Published:** 2025-07-11

**Authors:** Benedikt Hoeh, Jeremy Kwe, Fabian Schreiber, Tobias Hölscher, Katharina Boehm, Martin Baunacke, Roman Herout, Christian Thomas, Angelika Borkowetz

**Affiliations:** 1 Department of Urology, University Hospital Carl Gustav Carus, TUD Dresden University of Technology, Fetscherstraße 74, 01307 Dresden, Germany; 2 Department of Radiotherapy and Radiation Oncology, Faculty of Medicine, University Hospital Carl Gustav Carus, TUD Dresden University of Technology, 01307 Dresden, Germany; 3 Department of Urology, Universitätsmedizin Rostock, University Rostock, 18057 Rostock, Germany

**Keywords:** Prostate cancer, Adjuvant, Salvage, Biochemical recurrence, Multidisciplinary tumor board

## Abstract

**Background:**

In prostate cancer (PCa) patients treated with radical prostatectomy (RP), multidisciplinary tumor boards (MDT) issue recommendations to undergo adjuvant (aRT) or salvage radiotherapy (sRT). However, reliable data regarding the adherence rate to MDT recommendations and subsequently its impact on oncological outcomes are scarce.

**Methods:**

We retrospectively identified patients treated with RP within a certified prostate cancer center between 2012 and 2016, receiving an MDT recommendation to undergo adjuvant or salvage radiotherapy following RP due to adverse pathological features (non-organ confined disease, positive surgical margin, positive lymph node). Patients with a follow-up < 3 years were excluded. Among patients recommended to undergo aRT, adherence rate was calculated. Patients adherent vs. non-adherent to aRT recommendation were compared regarding biochemical recurrence (BCR), PSA failure following aRT or sRT, and cancer-specific (CSS) and overall survival (OS).

**Results:**

Of 802 patients, 408 (51.5%) were recommended to undergo aRT. Among those, 121 (30%) received aRT, whereas 287 (70%) were non-adherent to aRT-MDT recommendation. In multivariable logistic regression models, age, performance status and presence of adverse pathological features represented statistically significant predictors to undergo aRT. Rates of BCR (40.4 vs. 66.6%) as well as subsequent median time of PSA failure following radiotherapy (83 vs. 42 months) favored patients adherent to aRT recommendations (both *p* < 0.001). No differences were recorded in CSS and OS analyses regarding adherence to aRT recommendations.

**Conclusion:**

Solely a fraction of patients followed the recommendation to undergo aRT. Patients adherent to aRT recommendation demonstrated lower rates of subsequent PCa recurrences, albeit not (yet) translating into CSS/OS differences.

**Supplementary Information:**

The online version contains supplementary material available at 10.1007/s11255-025-04620-0.

## Introduction

In Germany, prostate cancer (PCa) is the most frequently diagnosed malignancy in men and accounts for the second leading cause of cancer-related death [1–3]. As a consequence of advances and progressive complexity of cancer treatments, designated certification programs were set up by the German Cancer Society (DKG) to ensure high-quality evidence-based therapy in accordance with current guidelines. In 2008, the DKG began certifying tertiary care center focusing on prostate cancer (‘Zertifiziertes Prostatakrebszentrum’) based on pre-specified criteria [4]. Since then, the number of DKG-certified tertiary care center focusing on prostate cancer has increased substantially and recent studies have demonstrated more favorable outcomes for patients treated within certified tertiary care centers compared to those treated in non-certified centers [3, 5]. Certified tertiary care centers are obliged to incorporate latest national and international guidelines to meet the DKG certification criteria. Furthermore, quality outcome measures need to be ascertained as well as defined structural requirements such as multidisciplinary tumor board (MDT) meetings issuing recommendations following local PCa treatment fulfilled [6, 7]. In patients treated with radical prostatectomy (RP) and high risk of PCa recurrence, data regarding adjuvant (aRT) vs. salvage radiotherapy (sRT) in case of biochemical recurrence (BCR) have been point of debate, and thus, MDT recommendations can be seen as an additional supportive guidance when decision is made regarding ongoing treatment algorithm [8, 9]. To date, contemporary real-world data regarding adherence to MDT recommendations in regard to RT (adjuvant vs. salvage) and its oncological impact among RP treated patients with high risk of BCR are limited [10, 11]. To address this void, we relied on a contemporary cohort of PCa patients treated with RP and subsequent MDT recommendation regarding radiotherapy modality. We hypothesized that only a minority of patients adheres to MDT recommendation. Furthermore, we hypothesized that adherence to MDT recommendation will likely translate into more favorable oncological outcomes.

## Material and methods

### Study population

After approval of the Ethic committee of the Technische Universität Dresden (EK53022014), we retrospectively identified PCa patients treated with RP who harbored at least one adverse pathology feature and a recommendation by the MDT following RP between 2012 and 2016. Adverse pathological feature was defined as non-organ confined disease (≥ pT3) and/or positive surgical margin (R1) and/or positive lymph node status (pN1). In accordance with the requirements to be accredited as a certified tertiary care center focusing on prostate cancer in Germany by the DKG, RP patients presenting at least one adverse pathological feature need to be discussed in MDT and subsequent recommendations were issued in regard to post-RP management. Within the study period, MDT recommendation to undergo aRT were issued to the present German S3-guidelines during this time period (version 3.0 and 4.0). Patients with a follow-up < 3 years were excluded.

### Statistical analyses

Descriptive statistics included the frequencies and proportions of categorical variables used in the analysis. Median values and interquartile ranges (IQR) were reported for all continuous variables. The Chi-square test was employed to evaluate the statistical significance of differences in proportions, while the t-test and Kruskal–Wallis test were used to analyze differences in distributions.

The statistical analyses consisted of the following steps: First, patients were stratified according to the MDT recommendations which consisted of either recommendation to undergo aRT or sRT in case of biochemical recurrence. In the first part of the analyses, patients with recommendation to undergo aRT were furthermore stratified according to adherent vs. non-adherent to the recommendation to undergo aRT. To test for a relation between adherence to MDT recommendation (aRT) and patient and tumor characteristics, uni- and multivariable logistic regression models were applied relying on the following variables: age (continuously coded), PSA at diagnosis (continuously coded), American Society of Anesthesiologists Physical Status Classification System (ASA) (I vs. II Vs. III), pN-Stage (pN0/x vs. pN1), pT-Stage (pT2 vs. ≥ pT3), surgical margin (R0 vs. R1/Rx), and pathological Gleason Grade Group (GGG1-3 vs. GGG4-5). Subsequently, comparison between patients adherent vs. non-adherent to aRT recommendation were performed in regard to biochemical recurrence and subsequent failure to radiotherapy relying on Kaplan–Meier plots. Moreover, additional exploratory endpoints consisted of cancer-specific and overall survival within this subgroup of patients.

In the second part of the analyses, patients with the recommendation to undergo sRT in case of BCR were selected and same statistical analyses as in the first part were, if applicable, repeated. R software environment for statistical computing and graphics (version 3.4.3) was used for all analyses [12].

## Results

### Descriptive characteristics of the overall study population

Overall, 802 patients qualified for analyses, of whom 9 patients (1.1%) were subsequently excluded due to inconclusive information regarding MDT recommendation. Among the residual 793 patients, 408 (51.5%) vs. 385 (48.5%) were recommended to undergo aRT vs. sRT (Supplementary Fig. 1).

### Characteristics of patients with aRT recommendation

Of 408 patients with the recommendation to undergo aRT, 121 (29.6%) vs. 287 (70.4%) adhered (and received aRT) vs. did not adhere, respectively. Patients who adhered to MDT recommendation were significantly younger (median age 67 vs. 69 years), harbored higher PSA at diagnosis (14 vs. 11 ng/ml), and better ASA status (Table 1). Moreover, patients adherent to MDT recommendation demonstrated less favorable histopathological features such as positive lymph nodes (69 vs. 41%) and higher rates of Gleason Grade Group (GGG) ≥ 4 at RP (61 vs. 39%), respectively (Table 1). In multivariable logistic regression analyses testing for an association between adherence to MDT recommendation and patient and tumor characteristics, age at surgery (OR: 0.95; 95%-CI: 0.93–0.99), ASA status (OR: 0.40; 95%-CI: 0.16–0.97), pN1-Stage (OR: 3.10; 95%-CI: 1.84–5.32), positive surgical margin (OR: 2.02; 95%-CI: 1.17–3.56), and GGG ≥ 4 (OR: 1.93; 95%-CI: 1.18–3.18) represented independent predictors to recommendations adherence (Table 2).

**Table 1:** Characteristics of 408 prostate cancer patients treated with RP and MDT-recommendation to undergo adjuvant radiotherapy stratified according to recommendation adherence between 2012 and 2016; all values are median (IQR) and frequencies (%)

MDT recommendation to adjuvant RT				
	Overall N = 408	Adherent, N = 121 (30%)^1^	Non-adherent N = 287 (70%)^1^	p-value
Age at surgery [years]	68 (62, 72)	67 (60, 71)	69 (63, 73)	0.003
PSA at diagnosis [ng/ml]	11 (7, 21)	14 (7, 27)	11 (7, 20)	0.074
ASA Physical Status				0.007
I.	39 (9.6%)	19 (16%)	20 (7.0%)	
II.	272 (67%)	82 (68%)	190 (67%)	
III.	94 (23%)	20 (17%)	74 (26%)	
pT-Stage				0.3
pT2	38 (9.3%)	10 (8.3%)	28 (9.8%)	
pT3	367 (90%)	109 (90%)	258 (90%)	
pT4	3 (0.7%)	2 (1.7%)	1 (0.3%)	
pN-Stage				<0.001
pN0	202 (50%)	37 (31%)	165 (58%)	
pN1	200 (49%)	84 (69%)	116 (41%)	
pNx	5 (1.2%)	0 (0%)	5 (1.7%)	
Surgical margin				0.10
R0	151 (37%)	38 (31%)	113 (39%)	
R1	206 (50%)	71 (59%)	135 (47%)	
Rx	51 (12%)	12 (9.9%)	39 (14%)	
Gleason Grade Group at surgery				<0.001
2	106 (26%)	24 (20%)	82 (29%)	
3	111 (28%)	23 (19%)	88 (31%)	
4	53 (13%)	18 (15%)	35 (12%)	
5	132 (33%)	55 (46%)	77 (27%)	
Surgical approach				0.5
Open	350 (86%)	106 (88%)	244 (85%)	
Robotic-assisted	58 (14%)	15 (12%)	43 (15%)	

ASA: American Society of Anesthesiologists Physical Status Classification System; MDT: multidisciplinary tumor board; RP: Radical prostatectomy; RT: Radiotherapy; IQR: Interquartile range

**Table 2:** Uni- and multivariable logistic regression models investigating adherence to MDT recommendation within patients treated with RP and recommendation to undergo adjuvant radiotherapy

	Univariable				Multivariable			
	OR	95%-CI		p-Value	OR	95%-CI		p-Value
		2.5%	97.5%			2.5%	97.5%	
Age at surgery	0.95	0.92	0.98	0.002	0.96	0.93	0.99	0.01
PSA at diagnosis	1.01	0.99	1.02	0.07	1.00	0.99	1.01	0.84
*ASA Physical Status*								
I	*Ref*				*Ref*			
II	0.45	0.23	0.90	0.02	0.44	0.20	0.94	0.03
III	0.28	0.13	0.63	0.002	0.40	0.16	0.97	0.04
*pN-Stage*								
pN0/pNx	*Ref*				*Ref*			
pN1	3.33	2.13	5.28	< 0.001	3.10	1.84	5.32	< 0.001
*pT-Stage*								
pT2	*Ref*				*Ref*			
pT3/4	1.20	0.58	2.67	0.64	1.13	0.50	2.71	0.77
*Surgical margin*								
R0	*Ref*				*Ref*			
R1	1.56	0.99	2.51	0.06	2.02	1.17	3.56	0.01
Rx	0.92	0.42	1.89	0.82	1.42	0.60	3.23	0.41
*Gleason Grade Group at surgery*								
1–3	*Ref*				*Ref*			
4–5	2.36	1.53	3.67	< 0.001	1.93	1.18	3.18	0.008

ASA: American Society of Anesthesiologists Physical Status Classification System; 95%-CI: 95% Confidence Interval; MDT: multidisciplinary tumor board; OR: Odds ratio

### Oncological outcomes according to aRT recommendation adherence

**Fig. 1:**
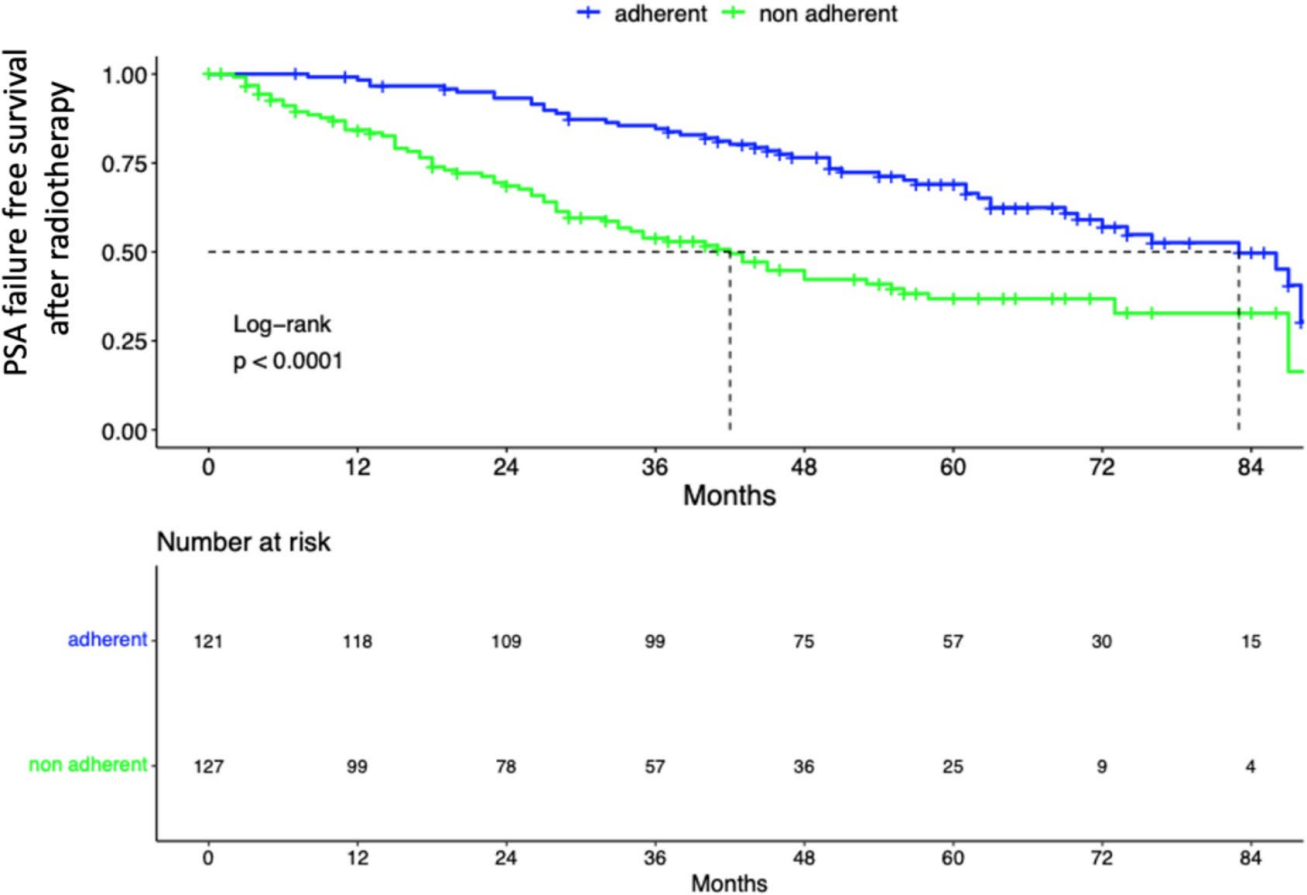
Kaplan–Meier depicting PSA failure-free survival following radiotherapy according to adherence to aRT recommendation adherence within 248 prostate cancer patients treated with RP;

**Fig. 2:**
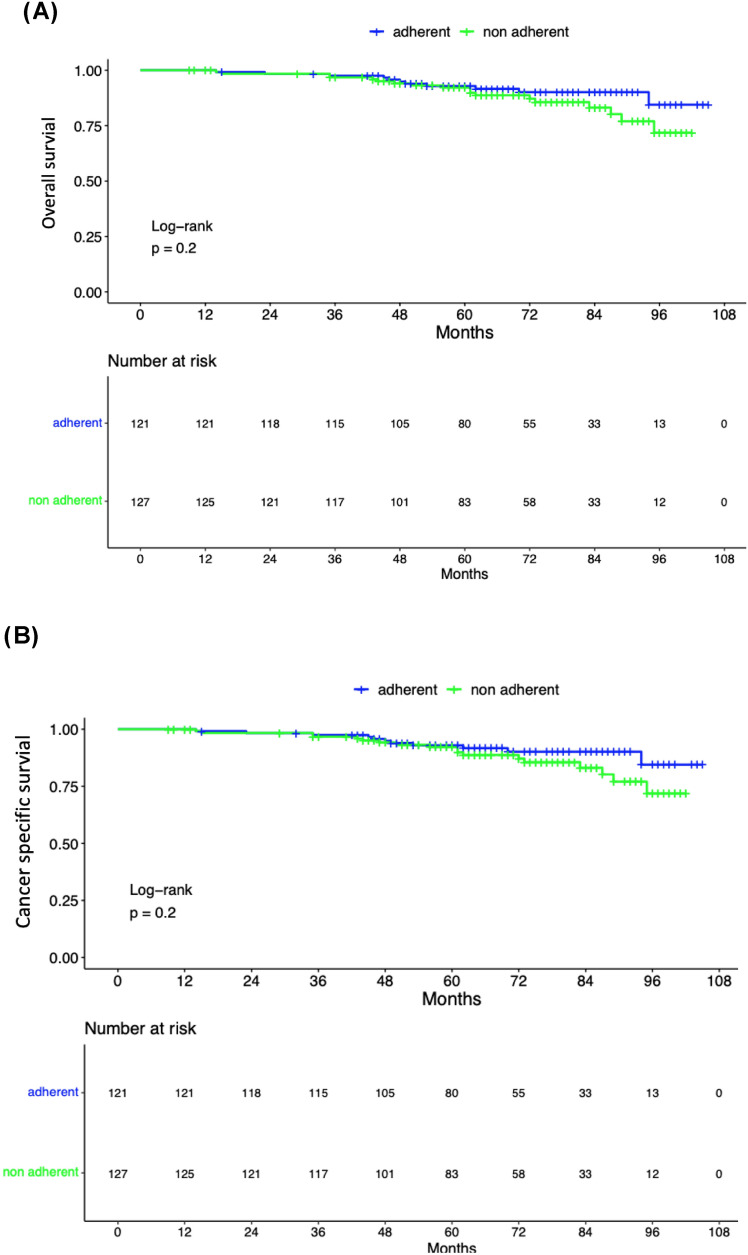
Kaplan–Meier curves depicting overall survival (**A**) and cancer-specific survival (**B**) according to aRT recommendation adherence within 248 prostate cancer patients treated with RP;

Among patients with aRT recommendation at a median follow-up time of 66 months (IQR: 49–82), BCR rates were 40.4% vs. 66.6% for patients adherent vs. non-adherent to aRT recommendation (*p* < 0.05; Supplementary Fig. 2). Among patients who did not adhere to aRT (*n* = 287) but subsequently developed a BCR (*n* = 191), 66.5% (*n* = 127) proceed to undergo sRT. Median time to PSA failure following radiotherapy was 83 (IQR: 70–not reached) vs. 42 (IQR:33–56) months for patients adherent to aRT vs. non-adherent to aRT, yet treated with sRT at time of BCR (Fig. 1, log-rank: *p* < 0.0001). No statistically significant differences were recorded in cancer-specific as well as overall survival analyses according to aRT recommendation adherence (Fig. 2A, B; log-rank: *p* = 0.2).

### Oncological outcomes of patients with sRT recommendation

Among the patients who were recommended to undergo sRT in case of PSA recurrence (Supplementary Table 1), 10 patients underwent aRT and were excluded from further statistical considerations (*n* = 375). At median follow-up time of 67 months (IQR: 55–84), 42.2% patients developed a biochemical recurrence of which 64.5% patients subsequently were treated with sRT. It is of note that no cancer-specific death was recorded within this subgroup of patients.

## Discussion

We hypothesized that the majority of RP treated patients adhered to MDT recommendation, and thus, adherence to MDT recommendation will translate into favorable oncological outcomes. We relied on a contemporary cohort of RP treated PCa patients at a certified tertiary care center focusing on prostate cancer and made some noteworthy findings.

In the first part of the analyses, we relied on the subgroup of RP patients recommended to undergo aRT (*n* = 402) according to MDT. Interestingly, yet simultaneously worrisome, solely a minority (*n* = 121; 30%) underwent aRT in line with MDT recommendations, whereas the majority of patients did not undergo aRT and therefore did not adhere to MDT (*n* = 287; 70%) recommendation. Even though that non-adherence to adjuvant therapy throughout malignancies of all entities is widespread and reported, the magnitude of non-adherence within the current study population is strikingly high [10, 13–15]. In line with the current findings, Knipper et al. reported a non-adherence aRT rate to MDT recommendation of 44.5% among 1140 RP patients and unfavorable pathological features between 2006 and 2015. Even though that the studies cannot directly be compared to each other due to the different study periods (2006–2015 vs. 2012–2016) and differences in inclusion criteria, the study by Knipper et al. together with the current findings underline the discrepancy of MDT recommendation and the adherence rate in a real-world setting.

When patient and tumor characteristics were compared between adherent and non-adherent patients, important differences were recorded (Table 1). Patients who followed MDT recommendation and underwent aRT were significantly younger, harbored a higher PSA at RP, and demonstrated a better health status evidenced by higher percentage of ASA I–II status compared to those patients who did not adhere to aRT. Moreover, tumor characteristics (pT-Stage, pN-Stage, surgical margin) were less favorable among patients adherent to adjuvant RT compared to those who did not adhere to aRT. Interestingly, pN1-Stage (OR: 3.10; 95%-CI: 1.84–5.32) as well as positive surgical margin (OR: 2.02; 95%-CI: 1.17–3.56) remained independent predictors to adherence among patients with the recommendation to undergo aRT, even after multivariable adjustment for other potentially confounding factors.

The discrepancy between aRT recommendation and actual adherence rate within this study cohort of RP patients can much likely be seen as a reflection of the ongoing debate and uncertainty regarding the optimal management of prostate cancer patients treated with RP and high risk of BCR. Even though that four prospective randomized trials have previously reported better cancer-control outcomes such as BCR survival or OS in aRT patients and especially those with adverse pathological criteria compared to observation following RP, potential severe toxic side effects and persistent toxicity after aRT must be taken into account when decision are made [16–19]. In order to reduce the probability of late lasting severe toxicity rates, sRT has been in the focus of ongoing investigations. Here, three published randomized-controlled phase III studies and one pooled meta-analyses compared aRT vs. sRT and reported no difference in five-year BCR-free survival or OS rates [9, 20, 21]. Interestingly, to take these findings into consideration, most recent updated guidelines are more restrictive in regard to recommend aRT [22, 23]. However and in contrast to these findings, data derived from retrospective analyses suggested that several risk factors were instead associated with worse cancer-control outcomes, favoring aRT in prostate cancer patients with at least one of the following risk factors: Gleason score 8–10, especially with R1 situation and pT3–4 and/or pN1 disease [8, 24, 25].

At a median follow-up time of 5.5 years, BCR rates (40.4 vs. 66.6%) were as expected significantly lower in patients who adhered to MDT recommendation (to undergo aRT) to those who did not adhere. Moreover, it is of note that a more favorable outcome in regard to PSA failure following radiotherapy (aRT or sRT) was recorded for those patients who initially adhered to aRT compared to those who did not adhere to aRT recommendation yet underwent sRT at time of BCR (Fig. 1). These findings suggest that when PSA failure to radiotherapy is considered as a primary endpoint, aRT results in more favorable outcomes compared to sRT. However, it should be emphasized that the current results are based on a retrospective cohort of RP treated patients. Moreover, despite those statistically significant and clinical meaningful differences in regard to PSA failure after radiotherapy, overall survival as well as cancer-specific survival analyses did not demonstrate differences between aRT and sRT, yet need to be interpreted under the light of extremely low rate of events for both endpoints.

Despite the noteworthy findings of the current study, some limitations need to be addressed. First and foremost, results need to be interpreted under the light of its retrospective, single-center nature and the limited study inclusion period. Nevertheless, within this study period, criteria for MDT recommendation to undergo either aRT or sRT remained unchanged. Moreover, no exact information regarding length of androgen deprivation therapy or the exact extent of the radiation to the pelvis were available. Due to the retrospective design of the current study, no exact data in regard to PSA thresholds and definition of BCR were at the discretion of the treating physicians. Additionally, staging modalities were at the discretions of the treating physicians and were subject to changes in guideline recommendations.

## Conclusions

Within this cohort of RP treated PCa patients with a high risk of PCa recurrence, solely 30% of patients followed the recommendation to undergo aRT. Patients adherent to aRT recommendation demonstrated lower rates of subsequent PCa recurrences. It is of note that these differences did not (yet) translate into differences in cancer-specific survival within the study cohort.

## Supplementary Information

Below is the link to the electronic supplementary material.

Supplementary file1 (DOCX 135 KB) Supplementary Fig. 1. Consort diagram of the study cohort stratified according to MDT recommendation.

Supplementary file2 (DOCX 140 KB) Supplementary Fig. 2. Kaplan–Meier depicting biochemical-free survival according to adherence to aRT recommendation within 408 prostate cancer patients treated with RP; 21 patients within the non-adherent subgroup were excluded due to missing information.

Supplementary file3 (DOCX 22 KB) Supplementary Table 1. Characteristics of 375 prostate cancer patients treated with RP and MDT-recommendation to undergo salvage radiotherapy; all values are median (IQR) and frequencies (%).

## Data Availability

No datasets were generated or analysed during the current study.
