# Supplementary material for: Adherence to post-therapeutic multidisciplinary tumor board recommendation and its influence on oncological outcomes in high-risk prostate cancer patients following radical prostatectomy
Source: Int Urol Nephrol. 2025 Jul 11;58(2):433–40. doi: 10.1007/s11255-025-04620-0 (PMC12864311; doi:10.1007/s11255-025-04620-0)
Supplement: Supplementary file 1 — Supplementary file1 (DOCX 135 KB) Supplementary Fig. 1. Consort diagram of the study cohort stratified according to MDT recommendation. [file 11255_2025_4620_MOESM1_ESM.docx]

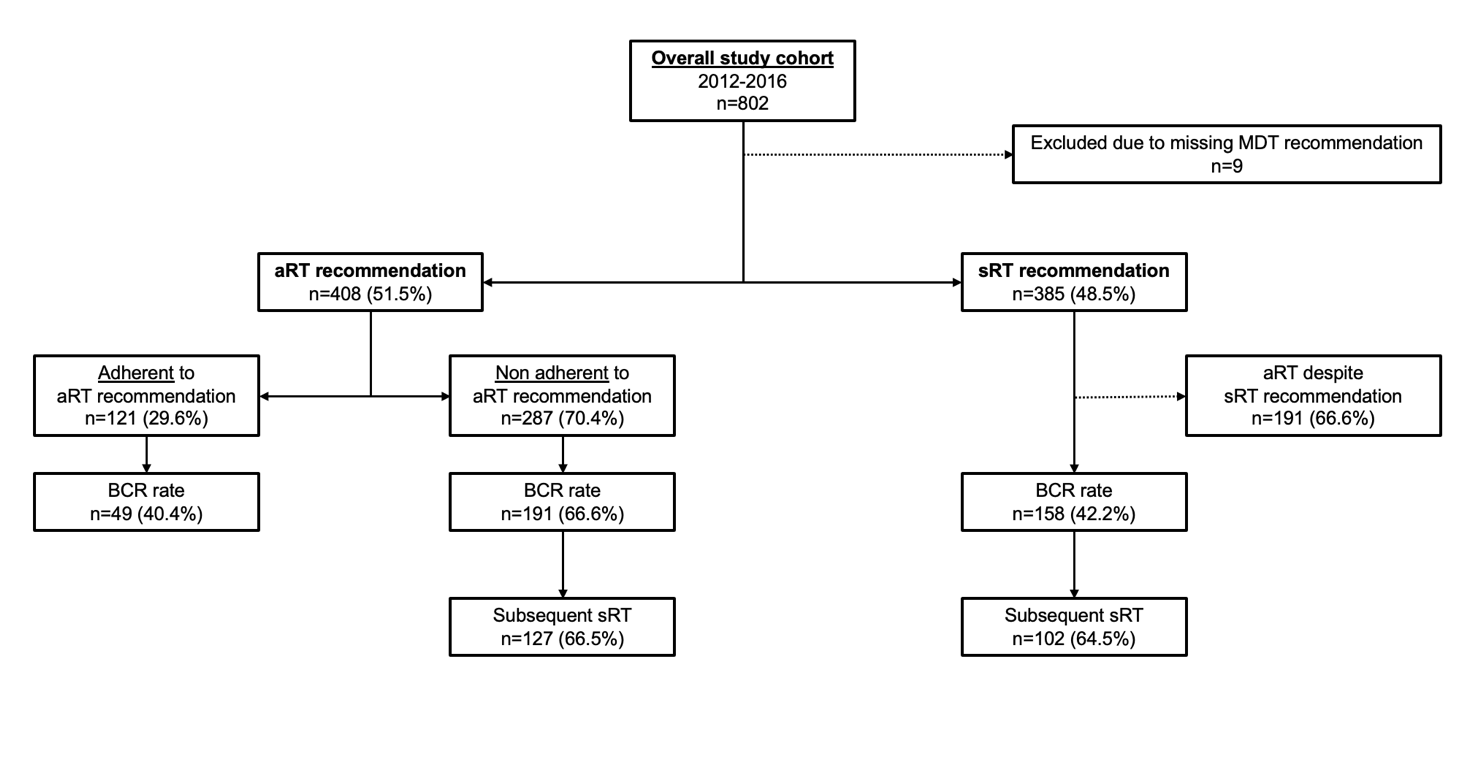


Abbreviations: aRT=Adjuvant radiotherapy; BCR=Biochemical recurrence; MDT=Multidisciplinary tumor board; sRT=Salvage radiotherapy;
