# Supplementary material for: Adherence to post-therapeutic multidisciplinary tumor board recommendation and its influence on oncological outcomes in high-risk prostate cancer patients following radical prostatectomy
Source: Int Urol Nephrol. 2025 Jul 11;58(2):433–40. doi: 10.1007/s11255-025-04620-0 (PMC12864311; doi:10.1007/s11255-025-04620-0)
Supplement: Supplementary file 2 — Supplementary file2 (DOCX 140 KB) Supplementary Fig. 2. Kaplan–Meier depicting biochemical-free survival according to adherence to aRT recommendation within 408 prostate cancer patients treated with RP; 21 patients within the non-adherent subgroup were excluded due to missing information. [file 11255_2025_4620_MOESM2_ESM.docx]

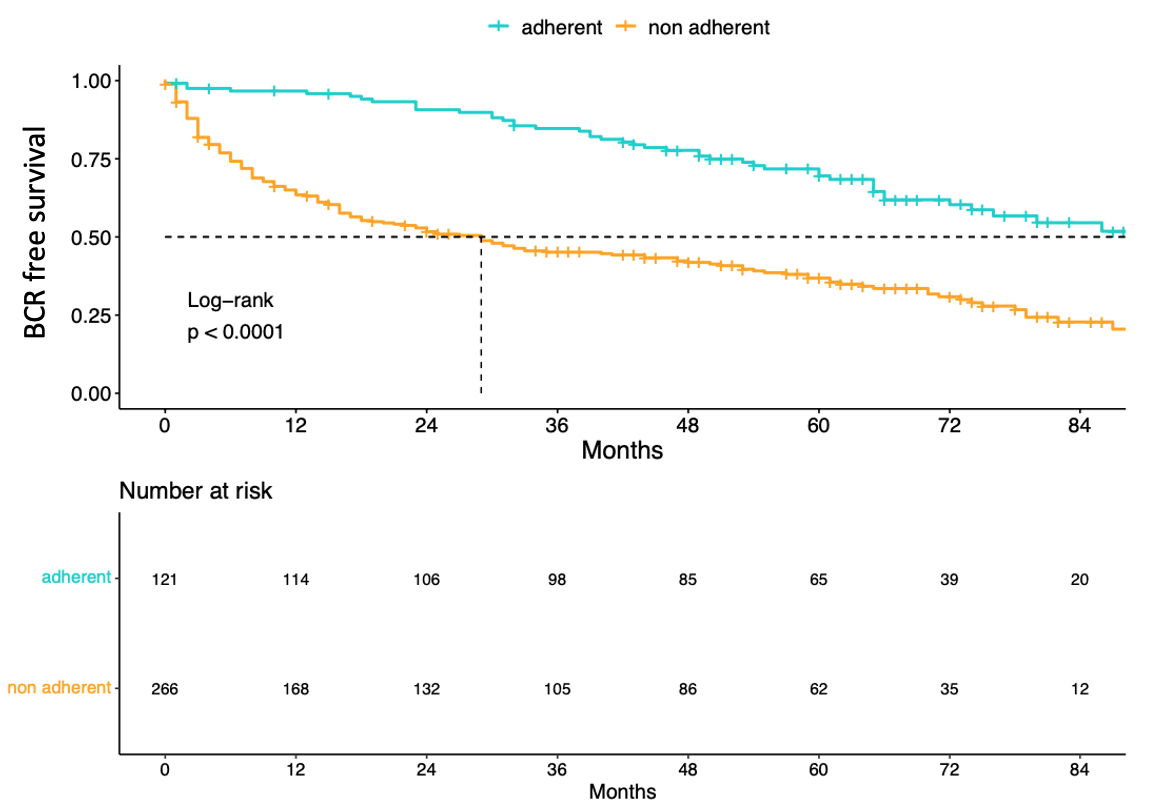


Abbreviations: aRT=Adjuvant radiotherapy; BCR=Biochemical reccurence; RP=Radical prostatectomy;
