# Supplementary material for: Adherence to post-therapeutic multidisciplinary tumor board recommendation and its influence on oncological outcomes in high-risk prostate cancer patients following radical prostatectomy
Source: Int Urol Nephrol. 2025 Jul 11;58(2):433–40. doi: 10.1007/s11255-025-04620-0 (PMC12864311; doi:10.1007/s11255-025-04620-0)
Supplement: Supplementary file 3 — Supplementary file3 (DOCX 22 KB) Supplementary Table 1. Characteristics of 375 prostate cancer patients treated with RP and MDT-recommendation to undergo salvage radiotherapy; all values are median (IQR) and frequencies (%). [file 11255_2025_4620_MOESM3_ESM.docx]

|  |  |  |
| --- | --- | --- |
|  | **N** | **Overall, N = 375** |
| Age at surgery [years] | 375 | 66 (61, 71) |
| PSA at diagnosis [ng/ml] | 361 | 8 (5, 11) |
| ASA Physical Status | 373 |  |
| I. |  | 40 (11%) |
| II. |  | 261 (70%) |
| III. |  | 71 (19%) |
| IV. |  | 1 (0.3%) |
| pT-Stage | 374 |  |
| pT2 |  | 132 (35%) |
| pT3 |  | 242 (65%) |
| pN-Stage | 372 |  |
| pN0 |  | 351 (94%) |
| pN1 |  | 5 (1.3%) |
| pNx |  | 16 (4.3%) |
| Surgical margin | 374 |  |
| R0 |  | 222 (59%) |
| R1 |  | 83 (22%) |
| Rx |  | 69 (18%) |
| Gleason Grade Group at surgery | 372 |  |
| 1 |  | 4 (1.1%) |
| 2 |  | 238 (64%) |
| 3 |  | 92 (25%) |
| 4 |  | 23 (6.2%) |
| 5 |  | 15 (4.0%) |
| Surgical approach | 374 |  |
| Open |  | 262 (70%) |
| Robotic-assisted |  | 112 (30%) |

Abbreviations: ASA= American Society of Anesthesiologists Physical Status Classification System; MDT=multidisciplinary tumor board; RP= Radical prostatectomy; RT=Radiotherapy; IQR=Interquartile range;
